# Supplementary material for: Characterization of Two New Multidrug-Resistant Strains of Mycobacterium smegmatis: Tools for Routine In Vitro Screening of Novel Anti-Mycobacterial Agents
Source: Antibiotics (Basel). 2019 Jan 2;8(1):4. doi: 10.3390/antibiotics8010004 (PMC6466533; doi:10.3390/antibiotics8010004)
Supplement: Supplementary file 1 [file antibiotics-08-00004-s001.pdf]

Supplementary Information

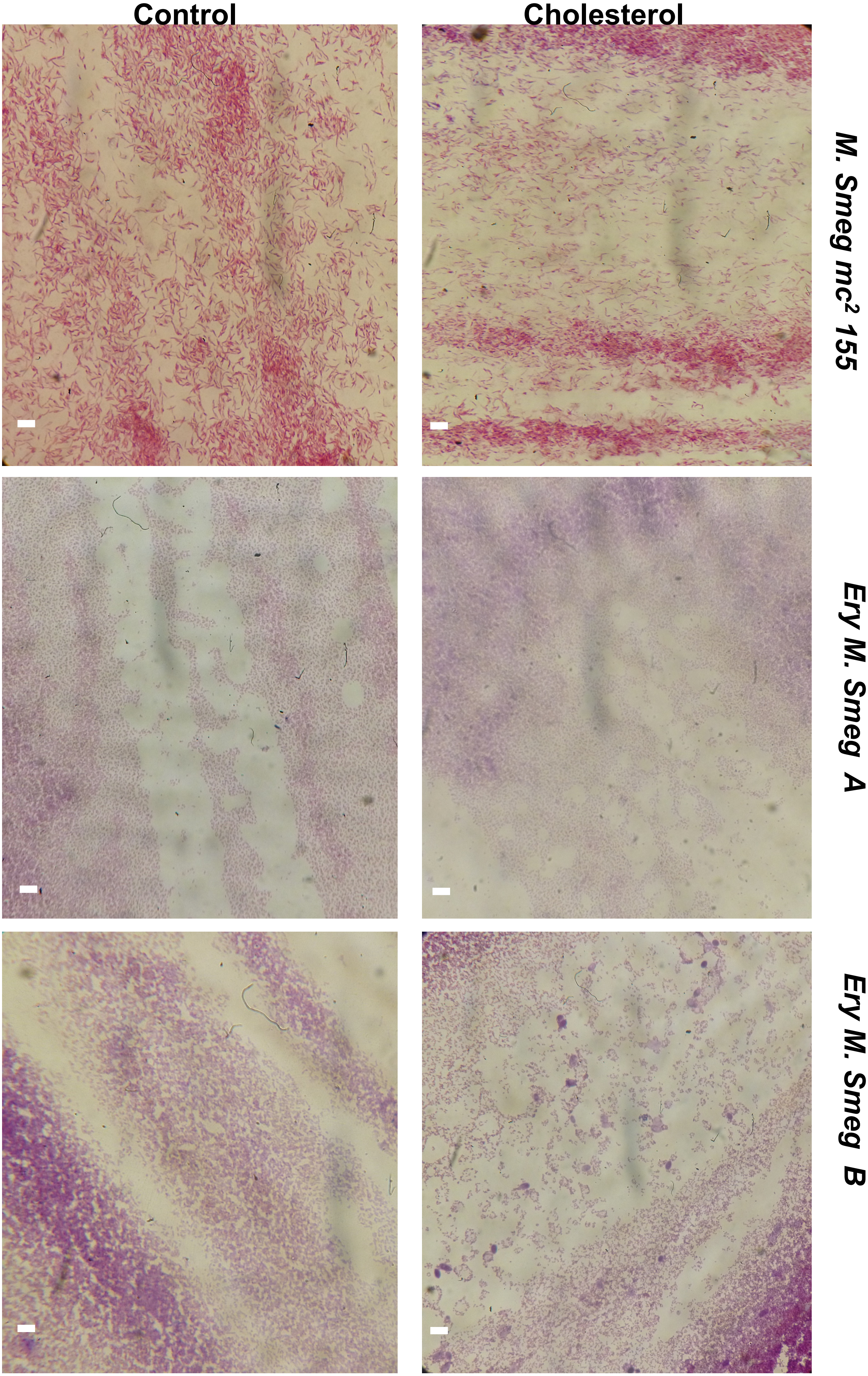

Figure S1:

Table S1. Optical Density of *M. smegmatis* as a measure of cell survival and proliferation during isolation of the MDR strains

Steps	Antibiotic	Optical Density		
		0hr	12hr	24hr
2	Tet 30μg	0.053	0.260	0.951
	Tet 150μg	0.049	0.201	0.864
	Ery 15μg	0.131	0.340	0.751
	Ery 75μg	0.098	0.297	0.698
	Strep 15μg	0.118	0.405	0.799
	Strep 75μg	0.143	0.345	0.912
4	Tet 30μg	0.101	0.071	2.285
	Tet 150μg	-	0.031	1.960
	Ery 15μg	0.114	1.026	1.998
	Ery 75μg	-	1.565	1.873
	Strep 15μg	0.127	1.977	2.106
	Strep 75μg	-	2.042	2.177
5	Tet 30μg	0.256	2.098	2.252
	Tet 150μg	0.114	2.004	2.137
	Ery 15μg	0.134	2.944	1.903
	Ery 75μg	0.111	2.339	2.411
	Strep 15μg	0.163	2.134	2.132
	Strep 75μg	0.193	2.204	2.171

Table S2: Amount of antibiotic in the paper discs used for the disc diffusion assay

Antibiotics	Abbreviation	Amount (µg)
Ampicillin	Amp	40
Amoxicillin	Amx	40
Vancomycin	Van	40
Isoniazid	INH	10
Ethambutol	Emb	10
Pyrazinamide	PZD	40
Moxifloxacin	Moxi	0.5
Rifampicin	Rif	10
Linezolid	Lin	5
Tetracycline	Tet	20
Chloramphenicol	Chlo	40
Erythromycin	Ery	40
Streptomycin	Strep	30

Table S3: Amount of antibiotic in the paper discs used as neighbours for the selected antibiotics

Antibiotics	Abbreviation	Amount (µg)
Ampicillin	Amp	40
Amoxicillin	Amx	40
Vancomycin	Van	40
Isoniazid	INH	10
Ethambutol	Emb	10
Pyrazinamide	PZD	40
Moxifloxacin	Moxi	0.5
Rifampicin	Rif	10
Linezolid	Lin	5
Tetracycline	Tet	20
Erythromycin	Ery	40
Streptomycin	Strep	30
Cycloserine	Cys	20
